# Integrated Transcriptomic and Metabolomic Insights into Flavor-Related Metabolism in Grape Berries Across Cultivars and Developmental Stages

**DOI:** 10.3390/metabo15100648

**Published:** 2025-09-29

**Authors:** Liping Huang, Linan Zhang, Min Wang, Yue Zhu, Zhili Xun, Xi Dai, Qifeng Zhao

**Affiliations:** 1 Pomology Institute, Shanxi Agricultural University, Jinzhong 030801, China; hlp-23@163.com (L.H.); wangmin@163.com (M.W.); zhy1012@sxau.edu.cn (Y.Z.); xzlgss@163.com (Z.X.); 2 College of Horticulture, Shanxi Agricultural University, Jinzhong 030801, China; 15735734695@163.com (L.Z.); daiqian@yy201902b.wecom.work (X.D.)

**Keywords:** grape berries, LC–MS metabolomics, transcriptome, fruit development, transcription factors

## Abstract

Background: Flavor quality in grape berries is shaped by complex metabolic and regulatory networks during development. Methods: In this study, we integrated transcriptomic and LC–MS-based metabolomic analyses to investigate three cultivars (‘Mei Xiangbao’, ‘Adena Rose’, and ‘Kyoho’) at two ripening stages. Results: A total of 491 differentially accumulated metabolites (DAMs) were identified, mainly lipids, organic acids, and heterocyclic compounds. Among them, 33 core metabolites, including LysoPCs, malic acid, and linalool derivatives, were closely linked to aroma, membrane remodeling, and polyphenol biosynthesis. Transcriptome integration revealed 29 transcription factors (TFs) such as AP2/ERF, MYB, and bHLH, which showed strong associations with key metabolites, suggesting their involvement in lipid remodeling and phenylpropanoid-related pathways. Conclusions: These results provide new insights into the molecular regulation of grape flavor metabolism and highlight candidate genes and metabolites for improving berry sensory quality.

## 1. Introduction

Flavor is a crucial determinant of fruit quality that strongly influences consumer acceptance and market value of table grapes and wine grapes alike [1,2]. In grape berries, flavor is a complex trait composed of a diverse array of volatile and non-volatile metabolites, including aromatic compounds, organic acids, sugars, polyphenols, and fatty acid derivatives [3,4]. These compounds not only contribute to sensory attributes such as aroma, taste, and mouthfeel but also reflect the physiological status and maturity of the fruit. Among them, volatile organic compounds (VOCs), such as C6 aldehydes and alcohols, terpenes, norisoprenoids, and esters, are particularly important for defining cultivar-specific aroma profiles [5,6]. Non-volatile components like malic and tartaric acid, glucose, fructose, and various phenolic compounds contribute to the balance of sweetness and acidity as well as to color, astringency, and antioxidant capacity [7,8]. The dynamic accumulation of these metabolites is tightly associated with grape berry development and ripening, and is known to vary across cultivars and environmental conditions. Given their biological and commercial significance, elucidating the molecular and metabolic basis underlying grape flavor formation has long been a focus in viticulture and enology research.

Advances in metabolomics have greatly enhanced our understanding of fruit flavor biosynthesis by enabling high-throughput profiling. In grapes, both gas chromatography-mass spectrometry (GC–MS) and liquid chromatography-mass spectrometry (LC–MS) have been widely used to quantify volatile aroma compounds, organic acids, amino acids, sugars, lipids, and phenylpropanoid derivatives across different cultivars and developmental stages [2,9]. These approaches have revealed complex networks of primary and secondary metabolites that contribute to varietal-specific and stage-specific flavor traits. Metabolomic studies have also shed light on important biochemical pathways involved in aroma formation, such as the lipoxygenase (LOX) pathway, phenylpropanoid pathway, and methylerythritol phosphate (MEP) pathway [10,11,12]. Lipid-derived volatiles, especially C6 and C9 compounds and lysophospholipids, have been highlighted as key aroma precursors or signaling molecules during grape ripening [13,14]. Furthermore, organic acids (malic and tartaric acid) and nitrogen-containing volatiles (pyrroles, pyridines) are increasingly recognized for their roles in balancing fruit flavor and shaping aroma complexity [15]. Despite these advances, metabolomic profiling alone often provides limited insight into the underlying regulatory mechanisms. Additionally, metabolite annotations, particularly for LC–MS-based untargeted analysis, remain challenging due to database limitations and low MS2 identification rates. Among the available analytical platforms, GC–MS and LC–MS offer complementary strengths in metabolite detection. Our previous work utilized GC–MS to characterize volatile aroma profiles in the same grape cultivars, revealing key differences in C6 aldehydes and terpene content across developmental stages [16]. However, LC–MS enables deeper insight into upstream metabolic precursors and signaling molecules, including lipid-derived intermediates, organic acid fluxes, and nitrogenous aroma precursors. In the present study, we applied untargeted LC–MS-based metabolomics to capture a more comprehensive picture of flavor-related metabolic networks beyond VOCs alone. Therefore, integrative omics approaches are needed to link metabolic shifts with regulatory gene networks and to identify key transcriptional regulators of flavor biosynthesis.

Flavor formation in grape berries is a highly regulated biological process involving complex metabolic pathways, including fatty acid derivatives, amino acid catabolism, and phenylpropanoid biosynthesis [17,18,19]. These processes are tightly controlled by developmental cues and environmental signals, mediated by changes in gene expression, enzymatic activities, and metabolite fluxes. While metabolomics reveals the end-point chemical profiles of flavor compounds, transcriptomics provides upstream insights into the gene regulatory mechanisms underlying these changes [20]. Recent studies have shown that integrated transcriptome-metabolome analysis enables the identification of key transcription factors (TFs), biosynthetic genes, and regulatory hubs that coordinate the accumulation of flavor-active compounds [21,22,23]. This approach has been successfully applied in both climacteric and non-climacteric fruits, but its potential in grapes, particularly for elucidating non-volatile components such as lipids, organic acids, and polyphenolic precursors, remains underexploited.

To better understand the formation of grape berry flavor, this study integrated LC–MS-based metabolomics and transcriptome analysis across three grape cultivars (‘Mei Xiangbao’, ‘Adena Rose’, and ‘Kyoho’) that differ in genetic background, flavor profile, and ripening dynamics. The novel early-ripening cultivar ‘Mei Xiangbao’ has garnered increasing attention due to its distinctive sensory properties. ‘Mei Xiangbao’ is a tetraploid interspecific hybrid, bred from a cross between ‘Adena Rose’ and ‘Kyoho’. It is characterized by soft flesh, a sweet taste, and a rich aroma profile dominated by rose- and strawberry-like notes, indicative of complex volatile biosynthesis during fruit maturation. This cultivar exhibits not only superior appearance and internal quality, but also strong disease resistance, wide ecological adaptability, and moderate yield potential. Due to its excellent comprehensive traits, ‘Mei Xiangbao’ was officially registered as a new grape variety by the Shanxi Provincial Crop Variety Approval Committee in December 2015 [24]. We focused on identifying key differential metabolites and flavor-related pathways, particularly those involving lipids, organic acids, and aroma precursors. By correlating metabolite profiles with DEGs and TFs, we aimed to reveal potential regulatory networks underlying flavor metabolism. This work provides new insights into the molecular basis of flavor diversity during grape ripening and offers valuable targets for breeding grapes with improved aroma and taste.

## 2. Materials and Methods

### 2.1. Plant Materials

Three table grape cultivars were used in this study: ‘Mei Xiangbao’ (M), ‘Adena Rose’ (A), and ‘Kyoho’ (J). All plant materials were grown in the grape breeding garden of the Pomology Institute, Shanxi Agricultural University (Taigu County, Jinzhong City, Shanxi Province, China). For each cultivar, samples were collected at two ripening stages based on skin coloration and aroma development: (1) an early stage, when berries had just begun to change color and developed a slight aroma; and (2) a late stage, when berries were fully colored and exhibited a stronger characteristic aroma. At each stage, three biological replicates were collected per cultivar. Each replicate consisted of approximately 15–20 healthy berries sampled randomly from multiple vines. After harvest, the berries were immediately peeled to remove skins and seeds, and only the flesh tissue was retained. The samples were then flash-frozen in liquid nitrogen and stored at −80 °C until further processing. In total, 36 grape flesh samples were obtained (3 cultivars × 2 stages × 6 replicates) for both LC–MS metabolomic analyses. The sample identifiers are as follows: ‘Kyoho’ (J1 for early stage, J2 for late stage), ‘Adena Rose’ (A1, A2), and ‘Mei Xiangbao’ (M1, M2).

### 2.2. Metabolite Profiling and DAM Selection

Widely targeted metabolome profiling was conducted using LC–MS/MS by Fujian Ailuoxuan Biotechnology Co., Ltd. (Fuzhou, China), on the same grape flesh (without skins) samples with six biological replicates per group. Chromatographic separation was performed on a reverse-phase C18 column, with water (0.1% formic acid) and acetonitrile as mobile phases under a gradient elution program. The mass spectrometer was operated in both positive and negative ionization modes, and MS/MS spectra were acquired using data-dependent acquisition. Metabolite identification confidence was evaluated according to the Metabolomics Standards Initiative (MSI). A subset of metabolites was confirmed by authentic standards (Level 1), while most were putatively identified by spectral matching against public and self-built databases (Level 2). Additional metabolites were annotated at the compound class level (Level 3), or remained unclassified despite having valid MS/MS spectra (Level 4). After preprocessing and peak alignment, metabolites were annotated by MS2 spectral matching against a public and self-built database. Differentially accumulated metabolites (DAMs) were screened using variable importance in projection (VIP) values from OPLS-DA models (VIP > 1) and fold change >2 or <0.5, with *p* < 0.05. Metabolites with consistent variation trends across all three cultivar comparisons were selected for further analysis. Superclass classification of DAMs was performed based on their chemical properties using MS2 annotations.

### 2.3. Metabolite Data Analysis

To visualize the overall metabolic differences among cultivars and ripening stages, principal component analysis (PCA) was conducted using normalized ion intensity data. The PCA scores plot revealed sample clustering patterns based on cultivar and developmental stage, reflecting metabolic differentiation during berry maturation. Metabolites were annotated and classified according to the Human Metabolome Database (HMDB) and the Kyoto Encyclopedia of Genes and Genomes (KEGG). The superclasses and subclasses of detected compounds were summarized, and the distribution of DAMs across different KEGG pathways was visualized. A bar chart was used to present the number of metabolites associated with each HMDB superclass, while KEGG classification was displayed by mapping DAMs to secondary pathway categories, grouped by KEGG’s top-level classification. Hierarchical clustering and heatmap visualization were performed to evaluate the variation in DAMs across samples. The normalized intensities of representative MS2-annotated metabolites were used, and Euclidean distance with Ward.D2 linkage was applied for clustering. KEGG pathway enrichment analysis was conducted using MetaboAnalyst and KOBAS. Significantly enriched metabolic pathways were identified by the hypergeometric test (*p* < 0.05), highlighting key pathways involved in aroma formation, lipid metabolism, and phenylpropanoid biosynthesis during grape berry development.

### 2.4. Transcriptomic Data Analysis

The RNA-seq data used in this study were obtained from our previously published work [16]. Briefly, total RNA from berry samples was extracted, and sequencing libraries were prepared and sequenced on the Illumina platform. Raw reads were quality-checked, aligned to the grape reference genome, and FPKM values were calculated using standard pipelines. Differentially expressed genes (DEGs) were identified using DESeq2 with thresholds of |log2FC| > 1 and adjusted *p*-value < 0.05. To identify transcription factors (TFs), DEGs were annotated against the *Vitis vinifera* reference genome and further classified using the Plant Transcription Factor Database (PlantTFDB). Candidate TFs belonging to families such as AP2/ERF, MYB, bHLH, bZIP, GATA, and MADS-box were subsequently analyzed for potential roles in regulating flavor-related metabolism.

### 2.5. Identification of Transcription Factors and Co-Expression Network

All DEGs were functionally annotated using the grape genome annotation and PlantTFDB to identify transcription factors (TFs). These TFs were considered candidate regulators involved in fruit aroma and metabolite biosynthesis. Pearson correlation analysis was performed between the expression levels of TFs and the contents of 29 representative DAMs. A threshold of |r| > 0.8 and *p* < 0.05 was applied to construct metabolite–gene correlation networks. Cytoscape v3.9.1 was used to visualize the interaction networks. Subnetworks highlighting specific TF–DAM associations in different compound classes were extracted and analyzed to reveal potential regulatory relationships.

## 3. Results

### 3.1. Overview of LC–MS Metabolomic Profiles

**Figure 1:**
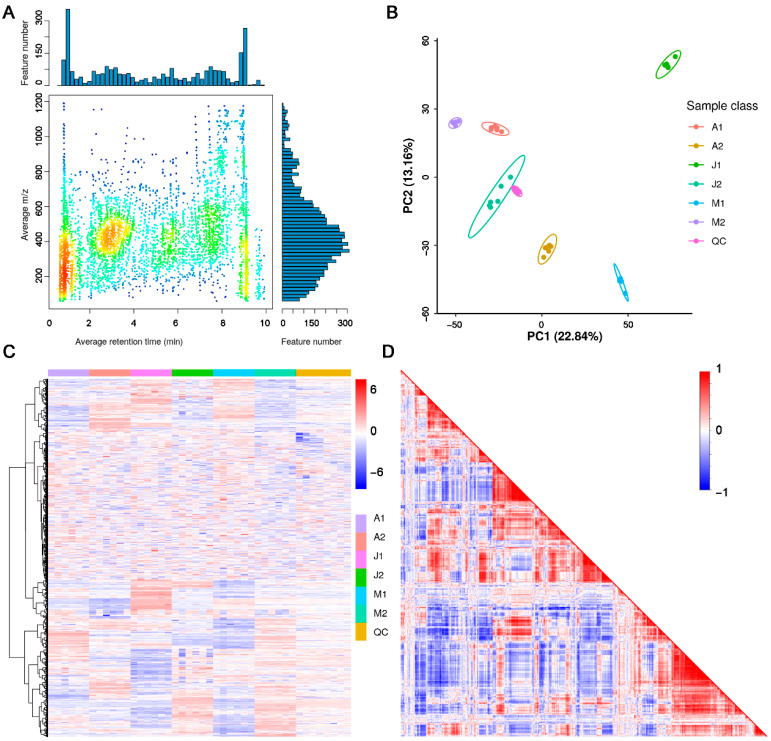
Overview of metabolite features detected by LC–MS in three grape cultivars across two ripening stages. (**A**) Distribution of metabolic features based on retention time and m/z ratio. Each dot represents a feature (metabolite ion), with color density indicating the abundance of features in the region; darker colors represent higher feature density. (**B**) Principal Component Analysis (PCA) plot based on normalized metabolite intensities. Each point represents one sample, and ellipses indicate 95% confidence intervals for each group. The PCA shows clear separation between cultivars and ripening stages, highlighting systematic differences in metabolite profiles. (**C**) Heatmap of all detected metabolites across all samples. Color represents scaled abundance (z-score) of each metabolite. Hierarchical clustering was performed using Euclidean distance and Ward’s method to visualize sample grouping and metabolite expression trends. (**D**) Metabolite correlation matrix heatmap. The plot displays pairwise Pearson correlation coefficients among all detected features. Red indicates positive correlation, blue indicates negative correlation, with color intensity reflecting the correlation strength.

To explore the dynamic metabolic changes underlying berry ripening and aroma development in grape, a widely targeted LC–MS-based metabolomics approach was employed to profile skin samples from the hybrid cultivar ‘Mei Xiangbao’ (M) and its parental cultivars ‘Adena Rose’ (A) and ‘Kyoho’ (J) at two developmental stages. A total of 10,700 metabolite features were detected (Table S1). The distribution of metabolite features in the m/z and retention time (RT) space revealed several high-density clusters (Figure 1A), suggesting the presence of distinct metabolite classes eluting in characteristic RT windows and m/z ranges. Principal Component Analysis (PCA) was used to examine the global variance across samples. The first two principal components (PC1 and PC2) accounted for 22.84% and 13.16% of the total variance, respectively, and clearly separated samples according to both cultivar and developmental stage (Figure 1B). In addition, a three-dimensional PCA incorporating PC3 (11.09%) was generated (Supplementary Figure S1), which together explained 47.09% of the total variance and provided improved visualization of cultivar- and stage-specific clustering. Notably, the hybrid ‘Mei Xiangbao’ formed a distinct cluster in both early and late stages, reflecting its unique metabolic profile. Hierarchical clustering and heatmap analysis further supported the distinct metabolic patterns among cultivars and developmental stages (Figure 1C). Most metabolites displayed stage-dependent changes in abundance, whereas a subset showed cultivar-specific accumulation patterns. Correlation analysis among all metabolite features revealed strong positive or negative correlations within metabolite groups, suggesting coordinated regulation of certain metabolic pathways during berry maturation (Figure 1D and Table S2). Together, these results indicate high data quality, strong biological repeatability, and significant genotype- and stage-dependent metabolic differences, providing a reliable foundation for downstream differential metabolite identification and functional interpretation.

### 3.2. Functional Classification and Pathway Enrichment of MS2-Annotated Metabolites

**Figure 2:**
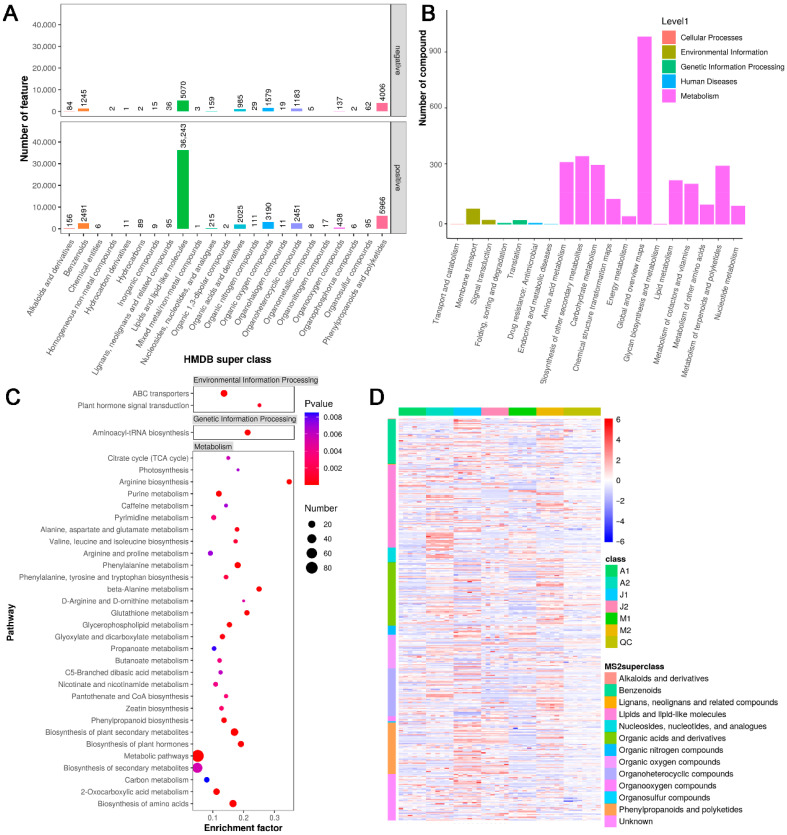
Functional classification and pathway enrichment of MS2-annotated metabolites. (**A**) HMDB super class classification of annotated metabolites. The *x*-axis represents different HMDB superclasses, while the *y*-axis indicates the number of features (top: all features; bottom: MS2-identified metabolites) falling into each category. (**B**) KEGG pathway classification at the Level 2 category. The *x*-axis shows secondary KEGG pathway terms, grouped by KEGG Level 1 categories (colored). The *y*-axis denotes the number of metabolites involved in each pathway. (**C**) KEGG pathway enrichment of MS2-identified metabolites. The *x*-axis shows the enrichment factor, and the *y*-axis lists enriched pathways. Dot size indicates the number of hits, and color represents the *p*-value of enrichment. (**D**) Heatmap of MS2-annotated metabolite intensities across all samples. The rows represent individual MS2-level metabolites, and columns are sample replicates. The metabolite classes (MS2 superclass) are indicated with color labels. Sample groups are shown in the top bar (A1, A2, J1, J2, M1, M2, QC).

To investigate the biochemical composition and potential functional roles of the identified metabolites, classification and enrichment analyses were performed based on MS2-annotated compounds (Figure 2). A total of 4096 metabolites were annotated at the MS2 level, and subsequently classified into HMDB superclasses based on their chemical properties (Figure 2A). Metabolite identification followed the MSI framework, with a subset confirmed by authentic standards and the majority annotated through spectral matching against reference databases. Among them, the dominant categories included lipids and lipid-like molecules (n = 896), organic acids and derivatives (n = 524), organonitrogen compounds (n = 358), and nucleosides, nucleotides, and analogues (n = 296). These classes encompass key compounds involved in fruit aroma formation, energy metabolism, and membrane lipid remodeling. Notably, lipid-related metabolites such as lysoPCs, triHOME, and fatty acid derivatives were prevalent, which may serve as precursors for aroma volatiles and signaling molecules during ripening. To gain insight into the biological pathways associated with the identified metabolites, KEGG pathway classification and enrichment analyses were conducted (Figure 2B). Based on KEGG Level 2 terms, most compounds were involved in metabolic pathways, particularly carbohydrate metabolism, amino acid metabolism, and lipid metabolism. These metabolic categories are closely related to ripening-associated aroma development and precursor biosynthesis. Further KEGG enrichment analysis revealed that significantly enriched pathways included phenylpropanoid biosynthesis, biosynthesis of secondary metabolites, plant hormone signal transduction, and glycerophospholipid metabolism (Figure 2C). These pathways are known to participate in the synthesis of aroma volatiles, stress-related responses, and membrane lipid modifications. A global heatmap of MS2-annotated metabolite intensities demonstrated distinct metabolomic profiles among the six sample groups (A1, A2, J1, J2, M1, M2), with clear clustering of biological replicates and separation between developmental stages (Figure 2D). In particular, lipid-related metabolites showed notable variation across ripening stages, supporting their key roles in aroma formation and fruit quality differentiation.

### 3.3. DAMs Among Cultivars and Developmental Stages

**Figure 3:**
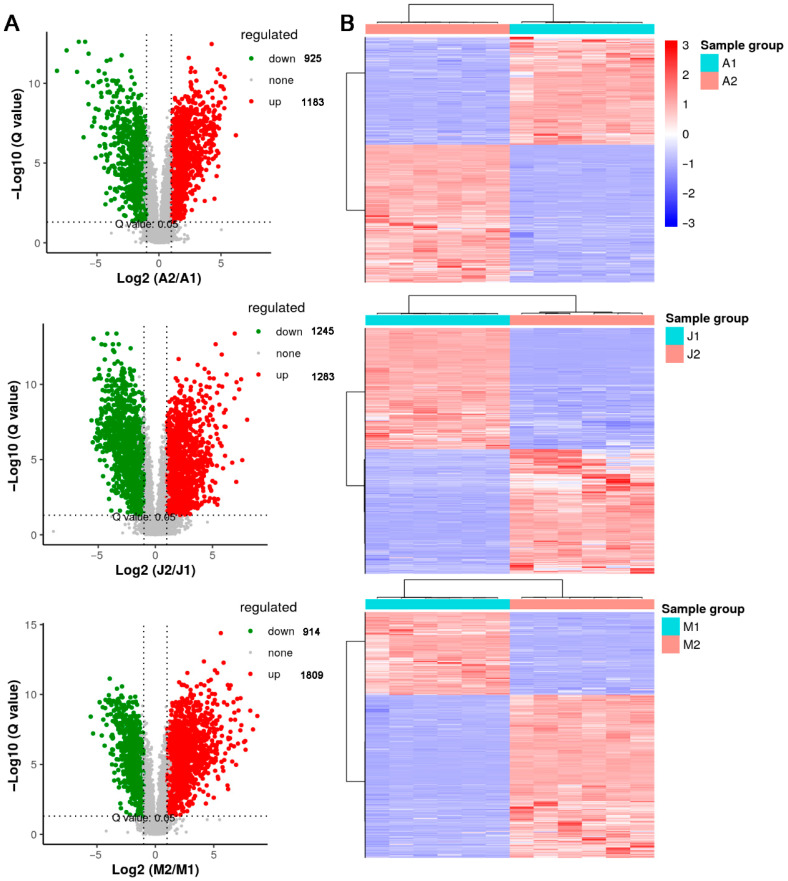
Identification and visualization of DAMs across developmental stages and cultivars. (**A**) Volcano plots illustrating DAMs between ripening stages within each cultivar: A2 vs. A1, J2 vs. J1, and M2 vs. M1. Red and green dots represent significantly upregulated and downregulated metabolites, respectively (threshold: |log2FC| ≥ 1 and Q-value < 0.05). (**B**) Heatmaps displaying the relative abundance patterns of DAMs across biological replicates in each pairwise comparison. The intensity values were normalized and scaled by row to highlight differences in metabolite profiles.

To characterize metabolite changes across different developmental stages and genotypes, pairwise comparisons were conducted based on LC–MS data (Figure 3, Supplementary Figures S2 and S3). The volcano plots illustrated a substantial number of DAMs between the early and late stages within each cultivar. Specifically, 2108 DAMs were identified in ‘Adena Rose’ (A2 vs. A1), 2528 in ‘Kyoho’ (J2 vs. J1), and 2723 in ‘Mei Xiangbao’ (M2 vs. M1) (Figure 3A). Among these, a considerable number were upregulated during ripening, suggesting active metabolomic reprogramming associated with aroma formation and flavor enhancement. The corresponding heatmaps (Figure 3B) revealed distinct clustering patterns between developmental stages, indicating reproducible metabolic shifts within each cultivar. Notably, ‘Mei Xiangbao’ exhibited a more extensive alteration in metabolite composition compared to its parents. Furthermore, inter-cultivar comparisons were performed at both early and late ripening stages (Supplementary Figures S1 and S2). At the early stage, M1 vs. A1 and M1 vs. J1 comparisons yielded 2436 and 2372 DAMs, respectively, including both up- and downregulated metabolites. At the late stage, M2 vs. A2 and M2 vs. J2 showed 2239 and 1402 DAMs, respectively, likewise reflecting both increases and decreases in metabolite abundance. These differences suggest that hybridization significantly reshaped the metabolic landscape of ‘Mei Xiangbao’, potentially contributing to its unique flavor profile. Collectively, these analyses uncovered substantial developmental and genotypic variation in metabolite profiles, providing a foundation for the subsequent identification of aroma-related and lipid-derived differential metabolites.

### 3.4. Functional Classification and Shared Features of DAMs

**Figure 4:**
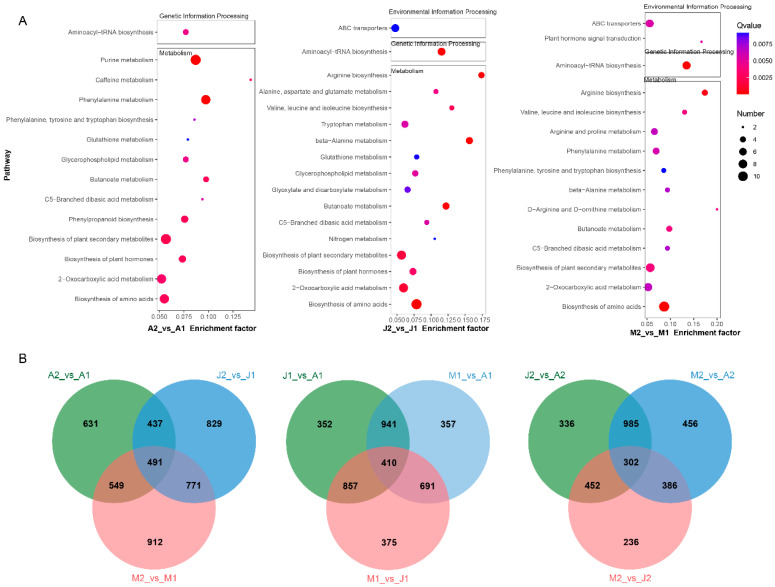
KEGG pathway enrichment and VENN analysis for DAMs across comparisons. (**A**) KEGG pathway enrichment analysis of DAMs in three intra-cultivar ripening comparisons (A2 vs. A1, J2 vs. J1, and M2 vs. M1). Pathways are ranked by enrichment factor, and dot size represents the number of DAMs enriched in the pathway. Dot color indicates Q-value. (**B**) Venn diagrams showing overlaps of DAMs among pairwise comparisons: stage comparisons (A2 vs. A1, J2 vs. J1, M2 vs. M1); early-stage genotype comparisons (J1 vs. A1, M1 vs. A1, M1 vs. J1); late-stage genotype comparisons (J2 vs. A2, M2 vs. A2, M2 vs. J2).

To gain insights into the biological functions of differential metabolites, KEGG enrichment analyses were performed on DAMs identified from the three ripening stage comparisons (A2 vs. A1, J2 vs. J1, M2 vs. M1). Enriched pathways were primarily associated with amino acid biosynthesis, secondary metabolite biosynthesis, and carbon metabolism (Figure 4A). For instance, phenylalanine metabolism, aminoacyl-tRNA biosynthesis, and glycerophospholipid metabolism were recurrent across all three cultivars, suggesting conserved biochemical shifts during grape berry maturation. ‘Mei Xiangbao’ (M) showed additional enrichment in pathways such as D-arginine and D-ornithine metabolism and plant hormone signal transduction, reflecting its unique hybrid genetic background and possibly its distinctive flavor traits. To further assess similarities and divergences among DAMs across comparisons, Venn diagrams were constructed (Figure 4B). In ripening-stage comparisons, 491 DAMs were commonly identified across A2 vs. A1, J2 vs. J1, and M2 vs. M1, indicating a core set of maturation-related metabolic changes (Table S3). In early-stage cultivar comparisons, 410 DAMs were shared among J1 vs. A1, M1 vs. A1, and M1 vs. J1, while in late-stage comparisons, 302 metabolites were commonly altered across J2 vs. A2, M2 vs. A2, and M2 vs. J2. These shared DAMs represent candidate metabolites responsible for genotype-specific flavor profiles and developmental regulation. Together, the enrichment and overlap analyses delineate the coordinated metabolic remodeling during grape berry ripening and underscore the influence of genetic background on aroma- and flavor-associated metabolite accumulation.

To further explore the biological implications of the 491 shared DAMs identified from multiple pairwise comparisons, we performed MS2-based metabolite annotation and selected 33 representative DAMs with high variable importance in projection (VIP) values (Table 1). These metabolites were categorized into distinct superclasses according to the MS2 annotation, including lipids and lipid-like molecules, organoheterocyclic compounds, organic acids and derivatives, and organic oxygen compounds. Among them, lipids and lipid-like molecules were the most abundant group (9 out of 33), including LysoPC 18:2, LysoPC 18:3, and 9,10,13-TriHOME. These compounds are not only key components of cellular membranes but also important precursors for aroma biosynthesis during grape ripening. For instance, 9,10,13-TriHOME, an oxidized derivative of linoleic acid, has been reported to contribute to fruity and green-like aromas in grape berries. The organoheterocyclic compounds (8 metabolites) included several bioactive compounds such as Alantolactone, Pyrrolidine, and Anileridine. This class of compounds is often involved in plant defense and signaling, and may also influence the biosynthesis of secondary metabolites like flavonoids and anthocyanins, thereby affecting berry color and quality traits. The group of organic acids and derivatives (4 metabolites), including D-(+)-malic acid and maleic acid, plays a central role in fruit acidity and flavor. Malic acid is a key organic acid in grapes, and its dynamic accumulation or degradation is closely linked to fruit development and consumer perception of taste. Lastly, organic oxygen compounds (5 metabolites), such as diacetyl, malondialdehyde, and linalool 3,6-oxide primeveroside, were also highlighted. These compounds are associated with both flavor development and oxidative stress responses. Linalool derivatives, for example, are important contributors to the floral and citrus-like aroma in Muscat-type grape varieties. These results indicate that the identified DAMs not only reflect significant metabolic shifts across developmental stages and cultivars but also relate closely to key phenotypic traits including flavor, color, and stress responses.

### 3.5. Identification of Differentially Expressed Transcription Factors (DETFs)

**Figure 5:**
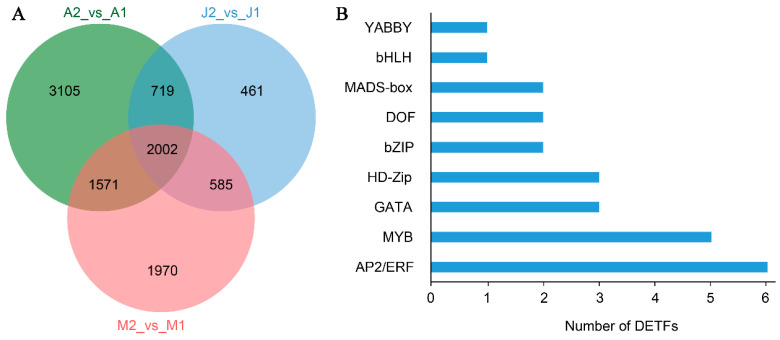
Identification of DEGs and DETFs during grape berry development. (**A**) Venn diagram showing the number of differentially expressed genes (DEGs) across three pairwise comparisons (A2_vs_A1, J2_vs_J1, M2_vs_M1). A total of 2002 common DEGs were shared among the three groups. (**B**) Classification of DETFs among the 2002 overlapping DEGs based on transcription factor families.

To further explore the transcriptional regulatory landscape associated with metabolite variation during grape berry development, we first identified differentially expressed genes (DEGs) from three key comparisons: A2_vs_A1, J2_vs_J1, and M2_vs_M1. A total of 2002 overlapping DEGs were found among these comparisons (Figure 5A and Table S4), representing a core set of genes consistently responsive to developmental changes across genotypes. Based on functional annotation and gene classification, 29 TFs were identified from the shared DEGs. These DETFs were classified into nine TF families, with the most abundant being AP2/ERF (6 genes), followed by MYB (5 genes), GATA and HD-Zip (4 genes each), and other TFs including bZIP, DOF, MADS-box, bHLH, and YABBY (Figure 5B). These TF families are widely known to regulate key biological processes such as hormone signaling, stress responses, and secondary metabolism. Notably, AP2/ERF and MYB families have been extensively linked to aroma formation, anthocyanin biosynthesis, and abiotic stress regulation in grape and other fruit crops. The presence of GATA and HD-Zip DETFs suggests possible involvement in light signaling and developmental transitions. These findings imply that the coordinated expression of specific transcription factors may underlie the observed metabolic shifts during ripening and contribute to varietal differences in fruit quality.

### 3.6. Correlation Analysis DETFs and Key Metabolites

**Figure 6:**
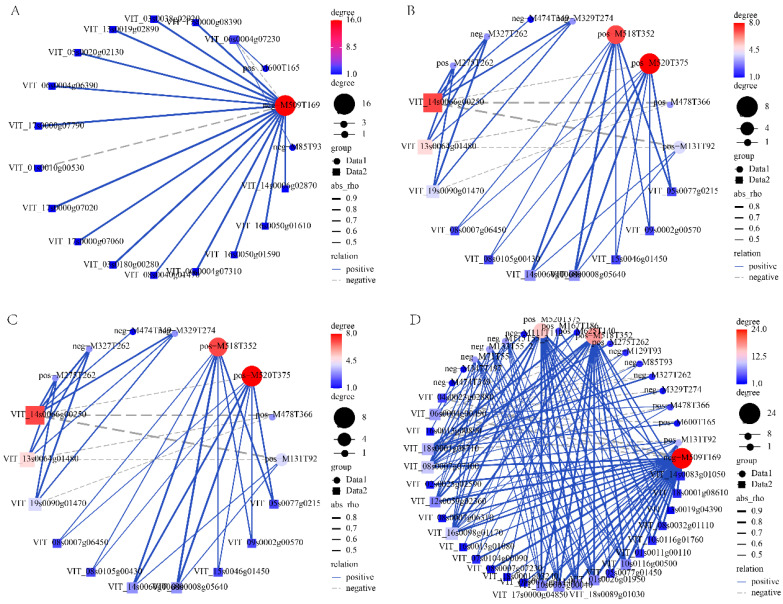
Correlation networks between DETFs and key DAMs. (**A**) Network showing DETFs–metabolite associations for organic oxygen compounds. (**B**) Network of DETFs correlated with lipids and lipid-like molecules. (**C**) Network involving DETFs and organoheterocyclic compounds. (**D**) Integrated network combining all 29 DETFs and 33 DAMs. Edge width indicates correlation strength (|r| > 0.8, *p* < 0.05); solid and dashed lines represent positive and negative correlations, respectively. Node size reflects degree centrality.

To further investigate the potential regulatory roles of candidate genes during grape development, we analyzed the expression of 29 DETFs and their associations with 33 key DAMs. These TFs, including members of the AP2/ERF, MYB, GATA, HD-Zip, MADS-box, and other families, were not necessarily differentially expressed across all comparisons but were filtered based on significant expression and correlation patterns. As shown in Figure 6A, VIT_18s0001g08610 (AP2/ERF) and VIT_18s0001g03240 exhibited strong positive correlations (r > 0.8, *p* < 0.05) with the volatile compound neg-M509T169 (Linalool 3,6-oxide primeveroside), which belongs to the organic oxygen compounds class. This compound is a key aroma-related metabolite, suggesting these AP2/ERF genes may contribute to aroma formation in ripening grape berries. In the lipid metabolism network (Figure 6B), VIT_16s0013g00890 (AP2/ERF) and VIT_07s0104g00090 (bHLH) were positively correlated with pos-M520T375 (LysoPC 18:2), neg-M327T262 ((9R,10S,12Z)-9,10-Dihydroxy-8-oxo-12-octadecenoic acid), and other lipid-like molecules. These lipids are associated with membrane remodeling and signaling processes during berry development. Regarding organoheterocyclic compounds (Figure 6C), several MYB (VIT_10s0116g00500, VIT_08s0007g06310) and VIT_04s0023g02880 (GATA) were linked with metabolites such as neg-M206T142 (1H-Indole-3-carboxylic acid) and pos-M233T170 (Alantolactone). These compounds are implicated in secondary metabolite biosynthesis and may relate to flavonoid or proanthocyanidin pathways important for berry pigmentation and antioxidant properties. In the integrated network (Figure 6D), VIT_18s0001g08610 (AP2/ERF) and VIT_10s0116g00500 (MYB) emerged as hub regulators, showing extensive associations with multiple DAMs across different classes. These results imply a coordinated transcriptional regulatory network involving TFs from various families potentially modulating lipid metabolism, aroma biosynthesis, and polyphenolic compound accumulation during grape ripening.

## 4. Discussion

To explore the metabolomic diversity during berry development, PCA clearly separated samples by both cultivar and two developmental stages, indicating distinct metabolic trajectories among ‘Mei Xiangbao’, ‘Adena Rose’, and ‘Kyoho’. Notably, the hybrid ‘Mei Xiangbao’ showed intermediate but unique profiles, reflecting a combination of parental traits and novel metabolite accumulation patterns. Functional classification of the DAMs based on HMDB and KEGG annotations revealed that lipids and lipid-like molecules constituted a major category, including phospholipid derivatives such as LysoPC 18:2, LysoPE 18:2, and 9,10,13-TriHOME (Figure 1 and Figure 2). These compounds are known precursors of aroma volatiles and play roles in membrane remodeling and signal transduction during ripening, such as in grape and tomato [25,26,27]. Other enriched categories such as organic acids and derivatives (malic acid, maleic acid) and organoheterocyclic compounds (pyrrolidine, indole-3-carboxylic acid) are tightly associated with acidity regulation, flavor balance, and nitrogenous aroma production. These results suggest that cultivar-specific regulation of lipid and amino acid metabolism contributes significantly to the flavor development in grape berries [14,28]. While several lipid derivatives identified here are widely recognized as precursors of C6/C9 volatiles, their direct conversion into aroma compounds was not tracked in this study. Future work integrating lipidomics with volatile profiling or isotopic labeling will be required to validate these biochemical pathways.

From the pool of 491 overlapping DAMs across the three cultivar comparisons, 33 representative metabolites were selected based on MS2-level annotation and high confidence (VIP > 1) (Tables S3 and Table 1). These compounds were further grouped into major superclasses, including lipids and lipid-like molecules, organoheterocyclic compounds, organic acids and derivatives, and organic oxygen compounds, each playing distinct roles in grape berry flavor development. Notably, several phospholipid derivatives such as LysoPC 18:2, LysoPC 18:3, and LysoPE 18:2 showed consistent downregulation during ripening in ‘Mei Xiangbao’ and its parents, indicating their consumption or conversion into volatile aroma compounds like hexanal and (E)-2-hexenal via the lipoxygenase (LOX) pathway [29,30]. These findings suggest their role as precursors of green and fruity aromas, contributing to cultivar-specific flavor attributes. In the class of organoheterocyclic compounds, metabolites like pyrrolidine and indole-3-carboxylic acid were more abundant in the later ripening stage, particularly in ‘Mei Xiangbao’. These nitrogen-containing volatiles are often associated with complex aroma notes such as roasted or nutty flavors, potentially enhancing the unique flavor identity of the hybrid [31]. Organic acids such as malic acid and maleic acid exhibited a decreasing trend during ripening, consistent with their role in modulating fruit acidity and taste balance [32,33]. Meanwhile, organic oxygen compounds including malondialdehyde and Linalool 3,6-oxide primeveroside were associated with oxidative stress and floral aroma formation, underscoring the multifaceted functions of these compounds during maturation [34,35]. Altogether, the identification and trend analysis of these representative metabolites highlight the coordinated metabolic shifts underlying aroma evolution, acidity adjustment, and fruit sensory enhancement in grape berries. These compounds not only serve as flavor-active molecules but also as metabolic indicators for cultivar differentiation and ripening stages.

To explore potential regulatory mechanisms behind metabolite accumulation, we investigated the transcriptomic patterns of differentially expressed TFs alongside key DAMs. A total of 25 TFs from families such as AP2/ERF, MYB, bHLH, HD-Zip, GATA, DOF, MADS-box, bZIP, and YABBY were identified among DEGs (Figure 5). These TFs are well known for their involvement in plant secondary metabolism, stress responses, and developmental regulation [36,37,38]. Correlation analysis revealed that several TFs displayed strong associations with specific classes of DAMs (Figure 6). For example, AP2/ERF TFs act as potential roles in regulating lipid metabolism and aroma precursor pathways. Similarly, AP2/ERF TFs have been implicated in regulating lipid-derived volatiles and aroma-related pathways in various fruit species, highlighting their potential roles in linking lipid metabolism to flavor formation [39]. MYB family members linking them to flavonoid biosynthesis and phenylpropanoid-related aroma. MYB TFs are well-documented regulators of anthocyanin accumulation and proanthocyanidin biosynthesis in grapevine [40,41]. In this study, their correlation with lignan derivatives suggests that MYB TFs may have a broader influence on phenolic metabolism, potentially contributing to phenolic diversity and affecting sensory attributes such as astringency and bitterness. A broader correlation network was constructed to integrate TFs and DAMs across major metabolite classes. The resulting TF–metabolite interaction map revealed potential central regulators such as VIT_06s0004g00490 (AP2/ERF) and VIT_01s0011g00110 (MADS-box), which exhibited extensive connections to both lipid-derived volatiles and nitrogen-containing aroma compounds. MADS-box TFs have been implicated in ripening regulation and secondary metabolism in fruits [42,43], suggesting that these hubs may integrate hormonal cues, metabolic fluxes, and textural changes to fine-tune flavor formation. Notably, the observed cultivar-specific patterns in TF–DAM correlations indicate that genetic background can shape transcriptional control of flavor traits. These hubs may coordinate multi-pathway regulation, linking lipid remodeling, organic acid metabolism, and aromatic amino acid derivatives during berry ripening. Moreover, these interactions appear to differ among cultivars, reflecting genetic background effects on transcriptional control. Such integrative insights are crucial for understanding the multi-layered regulation of flavor traits and provide molecular candidates for future breeding or gene-editing efforts. However, the perceptual contributions of these metabolites to grape flavor require further validation. Future work integrating metabolite profiling with sensory evaluation and rOAV analysis will be essential to directly link metabolic changes to sensory quality.

## 5. Conclusions

This study integrated transcriptomic and LC–MS-based metabolomic analyses to uncover flavor-related metabolic changes during grape berry development across cultivars. Key metabolites exhibited stage- and genotype-specific patterns, including lipids, organic acids, volatile heterocycles, and anthocyanins. Combined with differential expression and correlation analysis, several candidate transcription factors (AP2/ERF, MYB, and bHLH) were identified as potential regulators of flavor metabolism. These findings enhance our understanding of the molecular basis of grape flavor and provide valuable targets for future breeding and quality improvement.

## Figures and Tables

**Table 1:** List of 33 DAMs based on MS2 annotation.

ID	Metabolite (MS2)	Superclass(MS2)	A2_vs_A1	J2_vs_J1	M2_vs_M1	VIP (Max)
neg-M327T262	(9R,10S,12Z)-9,10-Dihydroxy -8-oxo-12-octadecenoic acid	Lipids and lipid-like molecules	down	up	up	2.278544
neg-M329T274	9,10,13-TriHOME	Lipids and lipid-like molecules	down	up	up	1.814051
pos-M131T92	Citraconic acid	Lipids and lipid-like molecules	up	down	down	1.416736
neg-M347T197	Foeniculoside VII	Lipids and lipid-like molecules	down	down	up	2.343692
pos-M520T375	LysoPC 18:2	Lipids and lipid-like molecules	up	down	down	3.387475
pos-M518T352	LysoPC 18:3	Lipids and lipid-like molecules	up	down	down	3.638893
pos-M478T366	LysoPE 18:2	Lipids and lipid-like molecules	up	down	down	2.335447
neg-M474T349	LysoPE 18:3	Lipids and lipid-like molecules	up	down	down	2.492346
pos-M275T262	Nandrolone	Lipids and lipid-like molecules	down	up	up	1.599161
neg-M206T142	1H-Indole-3-carboxylic acid	Organoheterocyclic compounds	down	down	up	1.887678
neg-M111T112	2-Furoic acid	Organoheterocyclic compounds	up	down	down	1.542474
neg-M129T93	4-Hydroxy-2-butenoic acid gamma-lactone	Organoheterocyclic compounds	up	down	down	3.307597
pos-M233T170	Alantolactone	Organoheterocyclic compounds	down	up	up	1.549641
pos-M353T274	Anileridine	Organoheterocyclic compounds	down	up	up	1.585172
neg-M179T144	Dihydroxyacetone (dimer)	Organoheterocyclic compounds	down	up	up	1.376759
pos-M86T80	Piperidine	Organoheterocyclic compounds	up	up	up	1.961697
pos-M72T54	Pyrrolidine	Organoheterocyclic compounds	up	up	up	1.652866
neg-M133T55_2	D-(+)-Malic acid	Organic acids and derivatives	up	down	down	2.619117
pos-M130T71	D-Pyroglutamic acid	Organic acids and derivatives	up	up	up	1.936217
neg-M115T55_2	Maleic acid	Organic acids and derivatives	up	down	down	2.572134
pos-M172T54	Tetrahydrodipicolinate	Organic acids and derivatives	up	down	up	1.995907
neg-M85T93	Diacetyl	Organic oxygen compounds	up	down	down	2.641908
neg-M509T169	Linalool 3,6-oxide primeveroside	Organic oxygen compounds	up	up	up	3.128103
neg-M71T55	Malondialdehyde	Organic oxygen compounds	up	down	down	2.262774
neg-M87T93	Malonic semialdehyde	Organic oxygen compounds	up	down	down	2.00654
pos-M268T72	Adenosine	Nucleosides, nucleotides	down	down	down	2.409461
pos-M664T64	beta-Nicotinamide adenine dinucleotide	Nucleosides, nucleotides	down	up	up	1.850993
neg-M262T188	Gemcitabine	Nucleosides, nucleotides	up	up	up	2.460595
pos-M318T324	Phytosphingosine	Organic nitrogen compounds	up	down	down	1.991469
pos-M203T41	Spermine	Organic nitrogen compounds	up	up	down	1.932743
pos-M625T140	Peonidin-3,5-O-di-beta-glucopyranoside	Phenylpropanoids and polyketides	down	up	up	2.273243
pos-M167T186	4-Ethyl-1,2-dimethoxybenzene	Benzenoids	down	down	up	2.080155
pos-M600T165	(7′R)-(+)-Lyoniresinol 9′-glucoside	Lignans, neolignans	down	up	up	2.050689

## Data Availability

The article contains all raw data. For further inquiries, please contact the corresponding author.

## Supplementary Materials

The following supporting information can be downloaded at https://www.mdpi.com/article/10.3390/metabo15100648/s1: Figure S1: 3D PCA of metabolite profiles based on LC–MS data. Figure S2: Pairwise comparisons between ‘Meixiangbao’ (M) and parental cultivars at the early stage; Figure S3: Pairwise comparisons between ‘Meixiangbao’ (M) and parental cultivars at the late stage. Table S1: Comprehensive metabolite abundance matrix and annotation for 10,700 detected features in grape berry skins from three cultivars (‘Mei Xiangbao’, ‘Adena Rose’, and ‘Kyoho’) across two ripening stages. Table S2: Pairwise Pearson correlation matrix of annotated metabolites. Table S3: List of 491 shared DAMs among three ripening-stage comparisons (A2_vs_A1, J2_vs_J1, M2_vs_M1). Table S4: Table S4 List of 2002 shared DEGs among three ripening-stage comparisons (A2_vs_A1, J2_vs_J1, M2_vs_M1).

## Abbreviations

The following abbreviations are used in this manuscript:

DAMs	Differentially accumulated metabolites
DEGs	Differentially expressed genes
DETFs	Differentially expressed transcription factors
GC–MS	Gas chromatography–mass spectrometry
HCA	Hierarchical cluster analysis
HMDB	Human Metabolome Database
KEGG	Kyoto Encyclopedia of Genes and Genomes
LC–MS	Liquid chromatography–mass spectrometry
OPLS–DA	Orthogonal partial least squares–discriminant analysis
PCA	Principal component analysis
QC	Quality control
rOAV	Relative odor activity value
TFs	Transcription factors
VIP	Variable importance in projection
