# Supplementary material for: Integrated Transcriptomic and Metabolomic Insights into Flavor-Related Metabolism in Grape Berries Across Cultivars and Developmental Stages
Source: Metabolites. 2025 Sep 29;15(10):648. doi: 10.3390/metabo15100648 (PMC12566481; doi:10.3390/metabo15100648)
Supplement: Supplementary file 1 [file metabolites-15-00648-s001.zip › Supplementary Figs_revised.pdf]

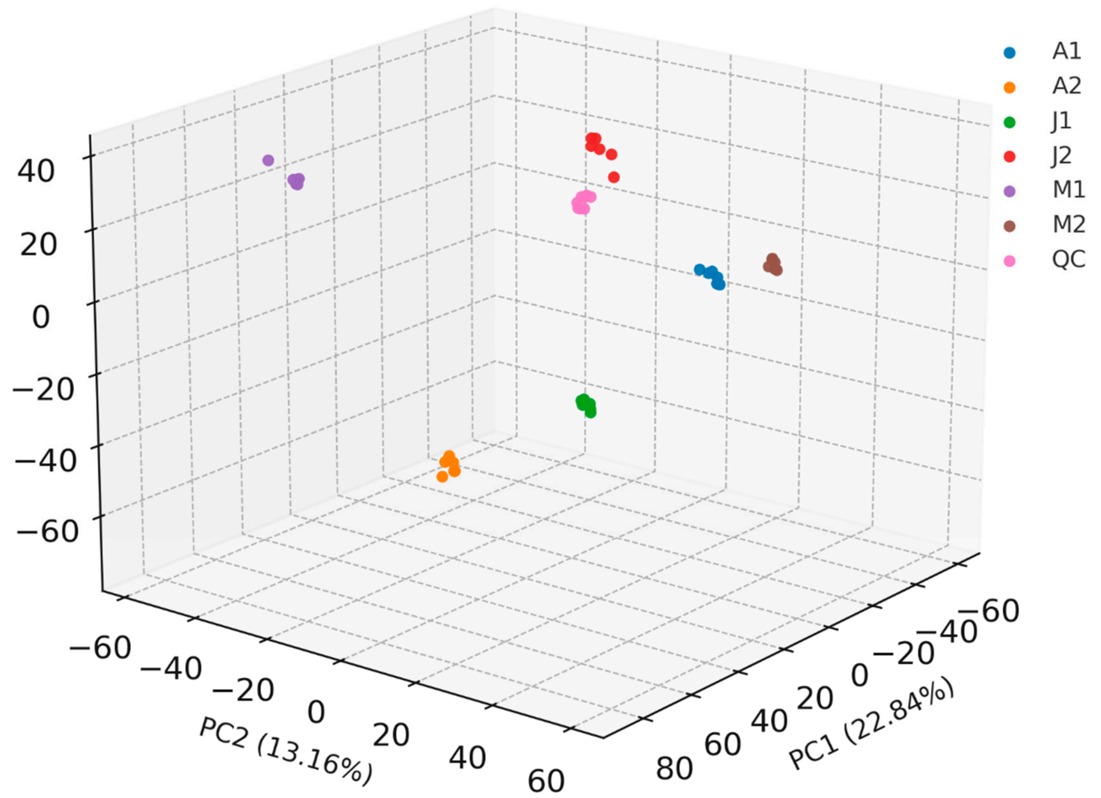

Supplementary Figure S1. Three-dimensional principal component analysis (3D PCA) of metabolite profiles based on LC–MS data. The first three components (PC1: 22.84%, PC2: 13.16%, PC3: 11.09%) together explained 47.09% of the total variance. Samples were separated by both cultivar and developmental stage, providing an improved visualization compared with the 2D PCA plot shown in Figure 1B.

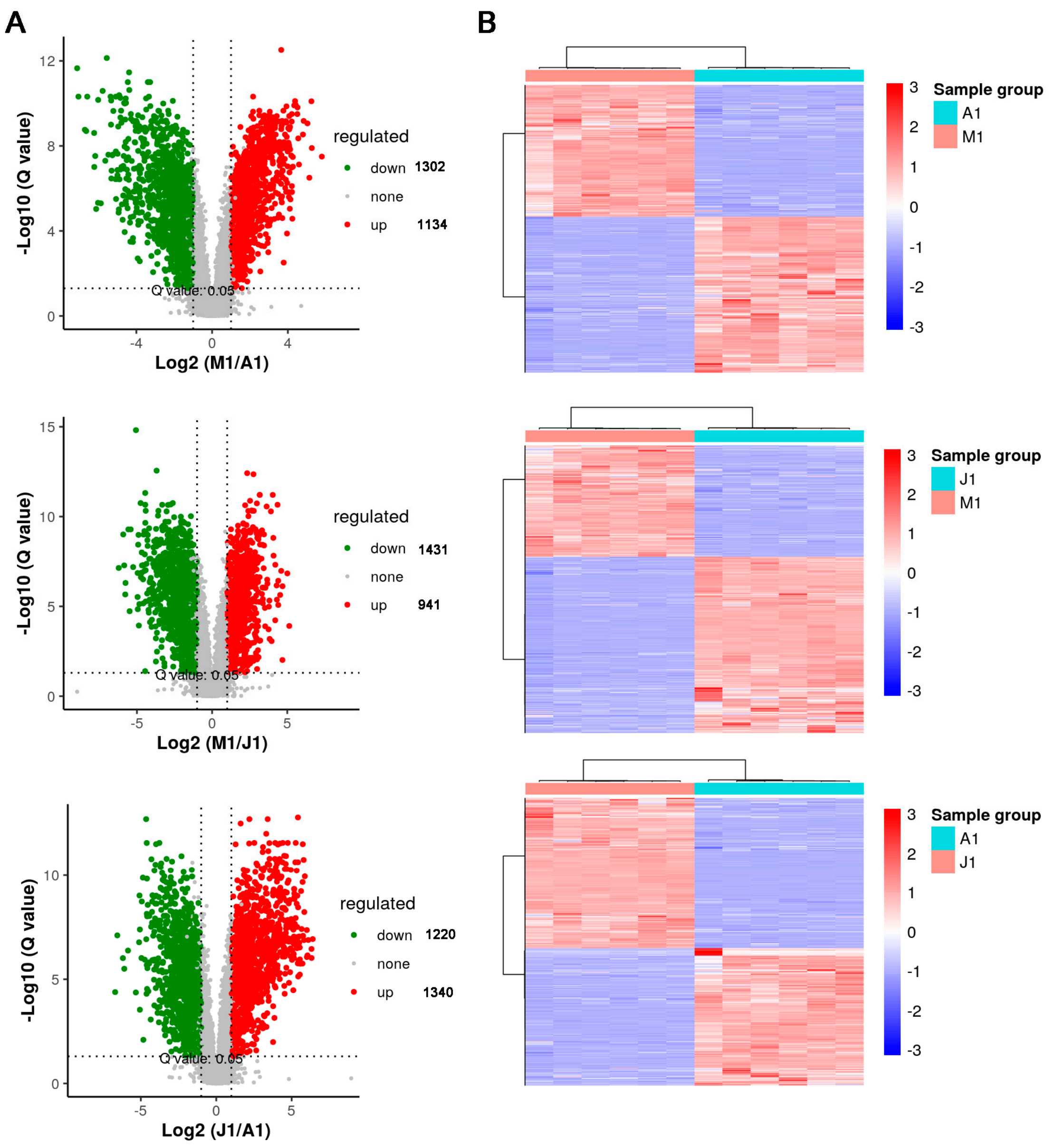

Supplementary Figure 2. Pairwise comparisons between 'Meixiangbao' (M) and parental cultivars at the early stage.

(A) Volcano plots of DAMs between M1 vs A1, M1 vs J1, and J1 vs A1.

(B) Corresponding heatmaps of DAMs for each comparison, visualizing clustering patterns of metabolite profiles.

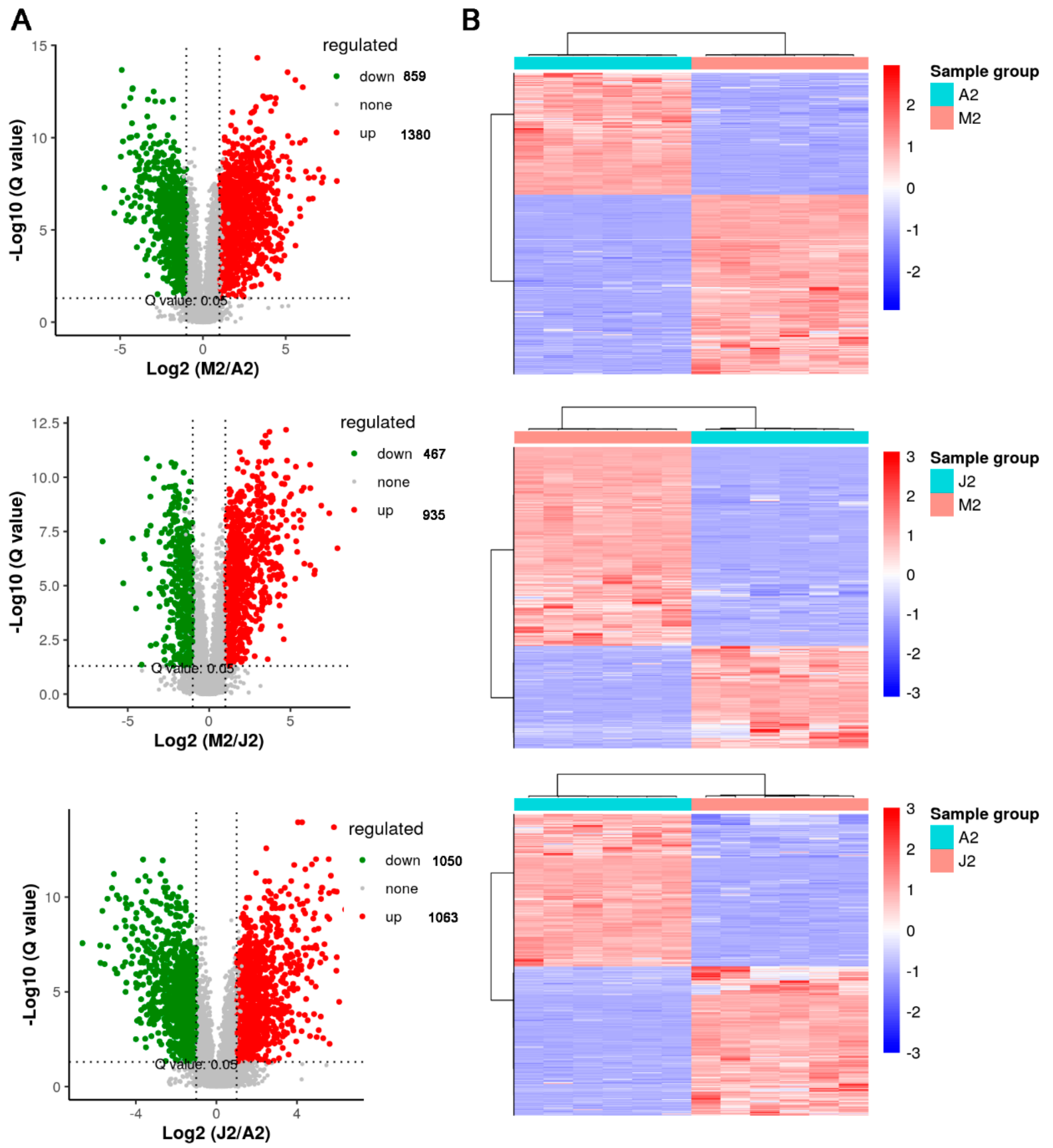

Supplementary Figure 3. Pairwise comparisons between 'Meixiangbao' (M) and parental cultivars at the late stage.

(A) Volcano plots of DAMs between M2 vs A2, M2 vs J2, and J2 vs A2.

(B) Corresponding heatmaps of DAMs for each comparison, based on row-scaled normalized intensity.
